# Multiple‐Dose Results from an Ongoing Phase 1 Study of Mivelsiran, an Investigational RNA Interference Therapeutic Targeting Amyloid‐Beta Precursor Protein for Alzheimer’s Disease

**DOI:** 10.1002/alz70859_097989

**Published:** 2025-12-25

**Authors:** Sharon Cohen, Simon Ducharme, Jared R. Brosch, Everard G.B. Vijverberg, Daniel J. Blackburn, Eric McDade, Alexandre Sostelly, Sandeep Chaudhari, Lynn Farrugia, Robert W Deering, Julia Shirvan, Catherine J. Mummery

**Affiliations:** ^1^ Toronto Memory Program, Toronto, ON Canada; ^2^ Montreal Neurological Institute, McGill University, Montréal, QC Canada; ^3^ Indiana University School of Medicine, Indianapolis, IN USA; ^4^ Alzheimer Center Amsterdam, Department of Neurology, Vrije Universiteit Amsterdam, Amsterdam UMC, Amsterdam Netherlands; ^5^ University of Sheffield, Sheffield, South Yorkshire United Kingdom; ^6^ Washington University in St. Louis, St. Louis, MO USA; ^7^ Alnylam Pharmaceuticals, Cambridge, MA USA; ^8^ Alnylam Pharmaceuticals, Inc., Cambridge, MA USA; ^9^ University College London, London, London United Kingdom

## Abstract

**Background:**

Single doses of mivelsiran, an investigational RNA interference (RNAi) therapeutic, have demonstrated robust amyloid‐beta precursor protein (APP) lowering in the CNS. We report additional interim safety and pharmacodynamic data in patients with early‐onset Alzheimer’s disease (EOAD) who received single ascending doses (SAD) and, for the first time, multiple doses of mivelsiran in the ongoing Phase 1 study (NCT05231785).

**Methods:**

Patients with EOAD (symptom onset <65 years of age, Clinical Dementia Rating global score 0.5 or 1.0, Mini‐Mental State Examination score >20) were randomized to a single intrathecal dose of mivelsiran 25–100mg or placebo for 6 months (plus up to 6 months follow‐up if needed for washout). After washout, patients could enter a separate multiple ascending dose (MAD) portion and receive open‐label mivelsiran. Presented here are data from a SAD cohort of mivelsiran 100mg and a MAD cohort of mivelsiran 50mg every 6 months (Q6M). Frequency of adverse events (AEs) and pharmacodynamics were primary and secondary endpoints, respectively.

**Results:**

Forty‐five patients were enrolled in SAD cohorts (as of 11/20/2024). Of those, 9 patients were randomized to mivelsiran 100mg or placebo (mean [SD] age, 64.1 [3.7] years; 33.3% male; 100% white). Most AEs were mild or moderate. Peak mean (SE) change from baseline in cerebrospinal fluid (CSF) soluble APP beta (sAPPβ) at Month 1 (─84.5% [1.3]) was largely sustained through Month 10 (─61.1% [2.8]).

After meeting washout criteria, 10 patients from SAD cohorts received mivelsiran 50mg Q6M (mean [SD] age, 59.9 [4.4] years; 70.0% male; 70.0% white). No serious or severe AEs were reported. At Day 15 after the first dose, mean (SE) change from baseline in CSF sAPPβ was ─63.7% (5.0); at Month 1 after the second dose, change was ─83.8% (2.3). Additional data will be presented.

**Conclusions:**

In this first report of multiple‐dose clinical data for a CNS‐targeting RNAi therapeutic, single and multiple doses of mivelsiran were generally well tolerated and continued to demonstrate robust, durable, dose‐dependent CSF sAPPβ reductions. Further sAPPβ lowering was observed after a second dose of mivelsiran 50mg. These results support further evaluation of mivelsiran in patients with Alzheimer’s disease or cerebral amyloid angiopathy.

